# Practical Implications of Ferromagnetic Artifacts in Low-field MRI-guided Radiotherapy

**DOI:** 10.7759/cureus.2359

**Published:** 2018-03-22

**Authors:** Olga Green, Lauren E Henke, Parag Parikh, Michael C Roach, Jeff M Michalski, H Michael Gach

**Affiliations:** 1 Radiation Oncology, Washington University School of Medicine, Barnes-Jewish Hospital

**Keywords:** mri artifact, mr-igrt, adaptive radiotherapy

## Abstract

Fractionated radiotherapy presents a new challenge in the screening of patients undergoing magnetic resonance imaging-guided radiotherapy (MR-IGRT). In our institution, patients are evaluated at the time of consult, simulation, and first fraction using a thorough MRI questionnaire identical to the one used for diagnostic radiology patients. For each subsequent fraction, the therapists are trained to inquire about any procedures the patient may have had between the last and current fractions. Patients are also advised to avoid food and fluid intake at least two but not beyond four hours prior to treatment. Despite these screening efforts, we have observed several non-permanent imaging artifacts that, while not harmful to the patient, prevent the accurate delivery of MR-IGRT when using online adaptive radiotherapy due to interference with the identification of relevant anatomy. Two such cases are presented here: (1) an imaging artifact due to iron-enriched breakfast cereal that precluded treatment for that day, and (2) an imaging artifact due to an iron-containing multivitamin that necessitated a creative solution to enable the accurate visualization of the area to be treated.

## Introduction

The first commercial system incorporating 0.35-T magnetic resonance imaging (MRI) with radiation therapy (MR-IGRT) became available in 2013 [1], with several other groups currently working on alternative systems at different field strengths [2-4]. The low field strength of this MR-IGRT configuration allows for fast, large field-of-view (FOV) volumetric imaging as well as real-time planar imaging at four frames per second (fps), with satisfactory image quality, as determined by standard testing protocols [5]. At our institution, the 0.35-T MR-IGRT system has been used to treat almost 700 patients to date [6], out of which almost 200 patients underwent real-time, online adaptive radiotherapy [7-8].

The current clinical workflow for MRI-guided adaptive radiotherapy includes consultation, computed tomography (CT), and 0.35-T MRI simulation, daily volumetric imaging, on-table manual recontouring, and plan re-optimization. Patients are asked to fill out an MRI questionnaire at the time of the initial consultation, which is then updated at the time of simulation and first fraction. The evaluation of the questionnaire at each of these stages is as thorough as the first time and generally adheres to the same procedures as for a diagnostic MRI study [9]. MR-IGRT therapists are thereafter responsible for inquiring about and evaluating the effect of any changes or medical procedures the patient may have undergone between fractions. Therapists also use a metal detector wand to ensure patient safety in the MR-IGRT vault at every fraction.

The majority of cases for online adaptive radiotherapy at our institution involve abdominal lesions, with the stomach, large bowel, small bowel, and duodenum being the primary organs at risk. Observation of daily anatomical changes and their impact on plan quality has led us to institute a two-hour minimum (four-hour maximum) restriction of fluid and food intake prior to a patient’s treatment in order to reduce this variability. However, some substances, even when ingested more than four hours prior to treatment, have been observed to cause magnetic susceptibility artifacts. Depending on the location and severity of the artifacts, treatment may be compromised due to the inability to accurately delineate the organs at risk or even visualize the target itself. Two example cases are presented in this work, both arising from the ingestion of iron – either in a vitamin supplement pill or in enriched breakfast cereal.

## Case presentation

Patient A is a 68-year-old man who presented with right upper-quadrant abdominal pain and was found to have unresectable intrahepatic cholangiocarcinoma. A diagnostic MRI demonstrated a 3.7x4.9 cm central mass in the caudate lobe of the liver, encasing the right hepatic artery and obliterating the left portal vein, with an enlarged adjacent portocaval lymph node. He enrolled in a clinical trial comprising hepatic arterial infusion with floxuridine, plus systemic gemcitabine and oxaliplatin. After three cycles, this was complicated by gastroduodenal arterial aneurysm requiring the discontinuation of therapy and surgical repair. Following repair, his disease remained localized and he was recommended to undergo hypofractionated ablative radiotherapy to the primary tumor and adjacent lymph node using MRI guidance and daily online plan adaptation. The planned prescription was 60 Gy in 15 fractions with concurrent capecitabine, utilizing daily online plan adaptation to protect the adjacent duodenum and stomach. A strict isotoxicity approach was planned such that if the goal planning target volume (PTV) coverage could not be achieved without organ at risk (OAR) constraint violation (e.g., duodenum), the coverage of the PTV was sacrificed to meet OAR constraints.

The patient successfully completed 14 fractions, with a re-optimized plan being used for eight of these due to an otherwise potential violation of the duodenum's or stomach's maximum point constraints. At the time of the last fraction, a large susceptibility artifact was observed on the pretreatment setup volumetric scan. This scan was acquired with a 17 sec three-dimensional (3D) true-fast imaging with steady-state precession (TrueFISP) sequence (echo time (TE): 2 ms, repetition time (TR): 4 ms, 1.6x1.6x3 mm resolution, 385 Hz/pixel, generalized autocalibrating partial parallel acquisition (GRAPPA): 2, 6/8 Fourier, flip angle: 60) [10], at exhale breath-hold, as per standard institutional procedure. The susceptibility artifact was located near the pylorus, partially obscuring the visualization of the duodenum and stomach, as well as the gross tumor volume (GTV) itself. The patient was removed from the MR-IGRT vault, as treatment was determined to be unsafe. Upon inquiry, the patient stated that he had indeed adhered to the “no food or drink” rule but had ingested a multivitamin pill shortly before treatment. The pill, having not yet passed through the digestive tract, was therefore still in his stomach. In order to proceed with therapy, the patient was asked to drink a cup of water, after which another imaging attempt was made.

**Figure 1:**
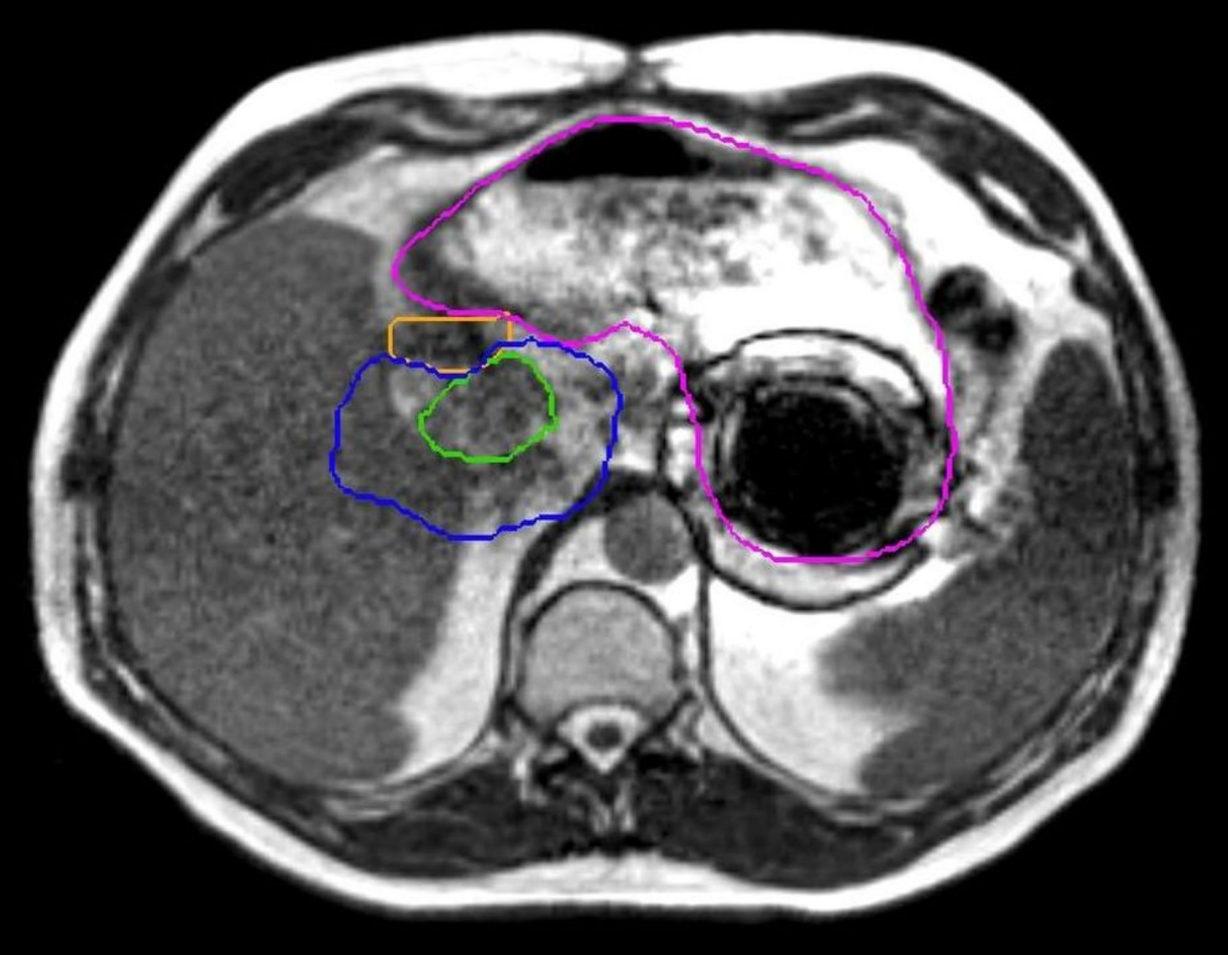
Vitamin pill artifact The GTV (green), the duodenum (orange), and the stomach (pink) were well visualized for the purposes of localization and adaptive reoptimization but only after the patient drank water to float the iron-containing pill to move the artifact away from the area of interest. The constraint isodose line (40 Gy, in blue) is seen to be well away from the artifact, as well. GTV: gross tumor volume

Figure 1 shows the subsequent location of the pill (judging by the artifact) after water intake. The image prior to this (with the artifact closer to the target and duodenum) is not available due to the MR-IGRT system not saving images in this workflow without treatment initiation. The water caused the pill to float to a different part of the stomach, which, in turn, improved the visualization of the GTV and adjacent anatomy, and the patient was successfully treated. It should be noted that the stomach was still imperfectly visualized, but as the planned dosimetric constraints (V40 Gy < 0.5cc) were all maximum point dose constraints to 0.5 cc volumes, visualization of the high-dose isodose regions of the re-optimized plan was adequate to ensure there was no possibility of the organ-at-risk violation.

Patient B is a 66-year-old man who initially presented with abdominal pain and weight loss and was found to have unresectable, locally advanced pancreatic adenocarcinoma. A pancreatic protocol CT scan at diagnosis showed a 5.2x2.8 cm hypoenhancing mass involving the pancreatic body and tail, with extensive vascular involvement, including the encasement of the portal-splenic confluence, abutment of the celiac axis, encasement of the common hepatic and left gastric arteries, and complete occlusion of both the splenic and superior mesenteric veins. The patient received six months of full-dose gemcitabine and abraxane chemotherapy without progression but remained unresectable. He was therefore recommended to undergo definitive-intent stereotactic MRI-guided online adaptive radiotherapy (SMART) with daily online plan adaptation and sagittal cine planar MRI gating. The goal prescription dose was 50 Gy in five fractions, subject to strict OAR constraints to minimize his risk of toxicity, including the possibility of excess dose to the duodenum, stomach, small, and large bowel. A strict isotoxicity approach was planned per the preceding case.

**Figure 2:**
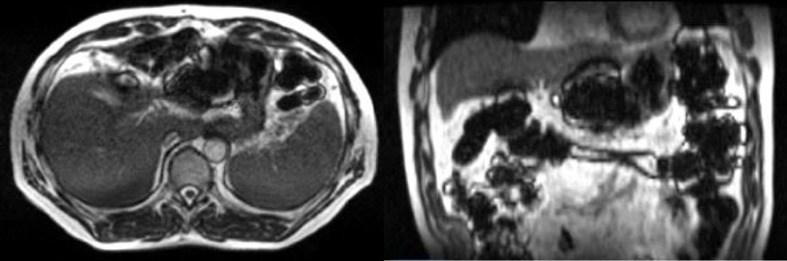
Grape-nuts artifact Transverse (left) and coronal (right) views of a patient’s abdomen a few hours after ingesting iron-fortified breakfast cereal. The susceptibility artifact present throughout the bowel prevented safe treatment for that day.

As with the first case, the patient was instructed to remain nothing-by-mouth for four hours prior to each treatment. The first two fractions were imaged, adapted, and treated without incident. However, after acquiring the 3D volumetric 17-sec TrueFISP scan (same parameters as Patient A) for the third fraction, a significant amount of artifact throughout the bowel was observed (Figure 2). The artifact prevented the accurate delineation of critical organs-at-risk, and therefore the treatment attempt was aborted. The patient was informed of this issue and an inquiry was made as to what he had ingested earlier in the morning. The answer – Grape-nuts (a product of Post Consumer Brands, Missouri, United States) – initially surprised the team. However, further investigation revealed that the Grape-nuts cereal contains a considerable concentration of iron that apparently became temporarily distributed through the patient’s bowel. The patient returned after a weekend break, subsequent 3D MRI revealed no artifact, and treatment commenced successfully.

## Discussion

**Figure 3:**
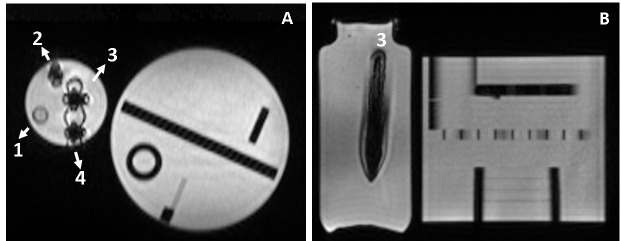
Grape-nuts in phantom Coronal (A) and axial (B) MRIs of Grape-nuts cereal (mixed with water or saliva) phantoms placed inside a 1 L bottle filled with sodium polyacrylate hydrogel. MRIs were acquired at 0.35 T using the 3D TrueFISP sequence. The 1 L bottle contained four 15 mL phantom tubes filled with: (1) water (control); (2) a small sample of unchewed Grape-nuts with water; 3) chewed Grape-nuts with saliva and water; and 4) unchewed Grape-nuts with water. The ACR phantom was placed next to the phantoms to load the body coil. The signal inside Phantom 3 is saturated (right) and the size of the phantom tube appears larger than its true dimension. Magnetic field lines appear as null bands in the coronal image for the three tubes containing Grape-nuts. MRI: magnetic resonance imaging; ACR: American College of Radiology

While the circumstances of the two artifacts presented above do not, in themselves, cause immediate harm to the patient from the MRI scans, they complicated the safe delivery of MR-IGRT and may have had a dosimetric impact if ignored. In the first case, the inability to visualize the area to be treated would have potentially resulted in mistreatment or excess dose to OARs if the patient was set up using surrounding organs or bony anatomy rather than the actual tumor, which was well-visualized for other fractions. However, a simple cup of water was able to move the artifact-inducing vitamin pill away from the treatment area. In the second case, the entire intestine was affected, and if an attempt was made to delineate it, it would surely have been inaccurate, erasing the potential dosimetric gains of online adaptive radiotherapy. To further investigate this unique scenario, a small quantity of Grape-nuts was crushed and placed in a phantom, which was then imaged using the 0.35-T MR-IGRT system’s TrueFISP volumetric sequence, as for patients. The results are shown in Figure 3, where it can be easily observed that compared to the original dimensions of the insert in the phantom, the Grape-nuts produced an artifact that significantly distorted the visualization of the insert. It should be stated that Grape-nuts are not the only potential culprit; a similar case was later observed with Cheerios cereal (General Mills) at our institution. Cheerios contains pulverized oats that are naturally high in non-heme iron (7.4 mg/cup), which is further fortified with additional non-heme iron. Indeed, any iron-fortified cereals and foods should be avoided during MR-IGRT when abdominal lesions are involved.

## Conclusions

MR-IGRT allows for a greater amount of information on the daily variation in patient soft tissue anatomy than ever before. Careful patient screening and fasting must be practiced to ensure safe and accurate imaging and radiation therapy. Patients must be educated by staff about the impact of consuming fortified foods and supplements on MR-IGRT and the importance of complying with exam preparation instructions.

## References

[1] Mutic S, Dempsey JF (2014). The ViewRay system: magnetic resonance-guided and controlled radiotherapy. Semin Radiat Oncol.

[2] Lagendijk JJ, Raaymakers BW, Raaijmakers AJE (2008). MRI/linac integration. Radiother Oncol.

[3] Keall PJ, Barton M, Crozier S (2014). The Australian magnetic resonance imaging-linac program. Semin Radiat Oncol.

[4] Fallone B, Murray B, Rathee S (2009). First MR images obtained during megavoltage photon irradiation from a prototype integrated linac‐MR system. Med Phys.

[5] Hu Y, Rankine L, Green OL (2015). Characterization of the onboard imaging unit for the first clinical magnetic resonance image guided radiation therapy system. Med Phys.

[6] Fischer-Valuck BW, Henke L, Green OL (2017). Two-and-a-half-year clinical experience with the world's first magnetic resonance image guided radiation therapy system. Adv Radiat Oncol.

[7] Henke L, Kashani R, Robinson CG (2017). Phase I trial of stereotactic MR-guided online adaptive radiation therapy (SMART) for the treatment of oligometastatic or unresectable primary malignancies of the abdomen. Radiother Oncol.

[8] Acharya S, Fischer-Valuck B, Kashani R (2016). Online magnetic resonance image guided adaptive radiation therapy: first clinical applications. Int J Radiat Oncol Biol Phys.

[9] Shellock FG, Kanal E (2008). MRI safety update 2008: part 2, screening patients for MRI. Am J Roentgenol.

[10] Bieri O, Scheffler K (2013). Fundamentals of balanced steady state free precession MRI. J Magn Reson Imaging.

